# Effect of Seven Newly Synthesized and Currently Available Oxime Cholinesterase Reactivators on Cyclosarin-Intoxicated Rats

**DOI:** 10.3390/ijms10073065

**Published:** 2009-07-07

**Authors:** Jana Zdarova Karasova, Jiri Kassa, Kamil Musilek, Miroslav Pohanka, Ladislav Novotny, Kamil Kuca

**Affiliations:** 1 Department of Toxicology, Faculty of Military Health Sciences, Trebesska 1575, 500 01 Hradec Kralove, Czech Republic; E-Mails: kassa@pmfhk.cz (J.K.); musilek@pmfhk.cz (K.M.); kucakam@pmfhk.cz (K.K.); 2 Center of Advanced Studies, Faculty of Military Health Sciences, Hradec Kralove, Czech Republic; 3 Department of Chemistry, Faculty of Sciences, J.E. Purkinje University, Horeni 13, 400 96, Usti nad Labem, Czech Republic

**Keywords:** acetylcholinesterase, butyrylcholinesterase, reactivators, oximes, cyclosarin

## Abstract

Seven new oxime-based acetylcholinesterase reactivators were compared with three currently available ones (obidoxime, trimedoxime, HI-6) for their ability to lessen cholinesterase inhibition in blood and brain of cyclosarin-treated rats. Oximes were given at doses of 5% their LD_50_ along with 21 mg/kg atropine five min before the LD_50_ of cyclosarin (120 ug/kg) was administered. Blood and brain samples were collected 30 minutes later. The greatest difference between acetylcholinesterase inhibition in blood of cyclosarin-treated rats was found after administration of HI-6 (40%), compared to 22% for trimedoxime and 6% for obidoxime. Only two of the seven newly synthesized oximes had any effect (K203 at 7%, K156 at 5%). Effective oximes against cyclosarin-inhibited plasma butyrylcholinesterase were HI-6 (42%), trimedoxime (11%), and K156 (4%). The oximes were less effective in brain than in blood, with reactivation values for HI-6 30% against acetylcholinesterase and 10% against butyrylcholinesterase. Values for newly synthesized oximes were less than 10% for K206, K269 and K203.

## 1. Introduction

Cholinesterase inhibitors based on phosphorus (OPI) are frequently used as insecticides in agriculture, industrial chemicals and some of these inhibitors with extremely high toxicity are called nerve agents. Nerve agents can be misused as chemical weapons or by terrorist groups. The mechanism of action of organophosphorus nerve agents (their toxicodynamics) is well known: irreversible inhibition of the enzyme acetylcholinesterase (AChE, EC 3.1.1.7) [1]. The inhibitory effect is based on phosphorylation or phosphonylation of serine hydroxyl group at the ester part of ChE active site of the enzyme [2].

The standard treatment of OPI intoxication usually consists of administration of anticholinergic drug (e.g. atropine) in combination with oximes. Anticholinergic drugs block effects of overstimulation caused by accumulated acetylcholine at peripheral muscarinic receptors while oximes, compounds with nucleophilic activity, repair the enzyme by dephosphorylation and restoring ChE’s activity [3]. However, the efficacy of commonly used oxime reactivators is still not sufficient [4].

**Figure 1.**
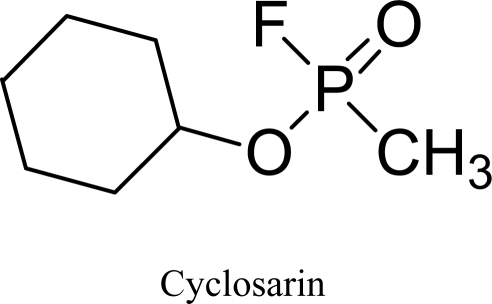
Chemical structure of cyclosarin.

Cyclosarin (GF agent; *O*-cyclohexyl-*N,N*-methyl phosphonofluoridate, Figure 1) belongs to a group of nerve agents or military importance that could be misused. The nerve agent cyclosarin has been examined to a minor extent compared to other nerve agents, e.g. sarin and soman. One reason is that this nerve agent was not regarded as a high priority chemical warfare agent until it was found to be stockpiled by Iraq in the early 1990s [5]. Commonly used bisquaternary reactivators (obidoxime, trimedoxime) of AChE are not able to counteract the toxic effect of cyclosarin because of their very low reactivating efficacy [6].

**Figure 2.**
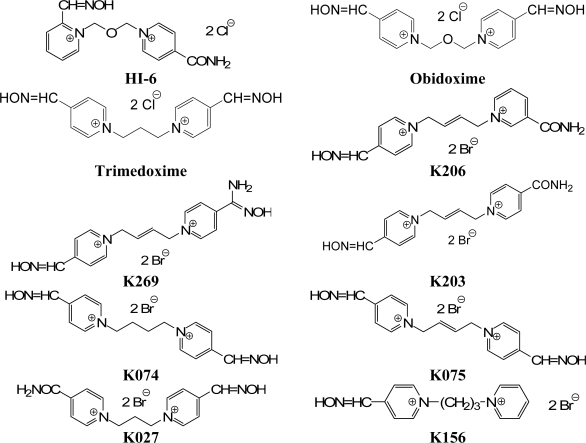
Chemical structures of tested AChE reactivators: Commonly used oximes were HI-6, obidoxime and trimedoxime. Newly synthesized AChE reactivators were K206, K269, K203, K074, K075, K027 and K156.

There are many important structural factors which can influence the reactivation potency of oximes [7,8]. The developments of new and more effective AChE reactivator still continue. For these reasons, seven new reactivators of cyclosarin-inhibited ChE (K206, K269, K203, K075, K074, K027, K156) have been synthesized [9,10] to increase the reactivating efficacy of antidotal treatment of poisoning by this nerve agent. The aim of this study was to evaluate *in vivo* antidotal effects of currently available oximes (obidoxime, trimedoxime, HI-6) and the seven newly synthesized oximes in combination with atropine (commonly used anticholinergic drug) in cyclosarin-poisoned rats (Figure 2). Syntheses as well as analyses of these reactivators were published formerly [9–14].

The other aim of this study was to compare antidotal effects of these compounds against cyclosarin-inhibited butyrylcholinesterase (BChE; EC 3.1.1.8). BChE is in plasma and also in brain. These *in vivo* data could be useful for preparation of an effective pretreatment therapy including administration of pseudo-catalytic bioscavenger.

## 2. Results and Discussion

No symptoms of intoxication are manifest if AChE activity is decreased by about 20–30 % compared to normal AChE activity. The depression about 30–50 % is accompanied by muscarinic symptoms of intoxication. The subsequent depression about 50–70 % of original AChE activity is characterized by muscarinic, nicotinic and also central symptoms. Finally, inhibition under a 20 % limit leads to death of the intoxicated organism. If reactivation of inhibited AChE is considered, increase in reactivation to more than 10 % may save the life of intoxicated organism and can reduce toxic symptoms.

**Figure 3.**
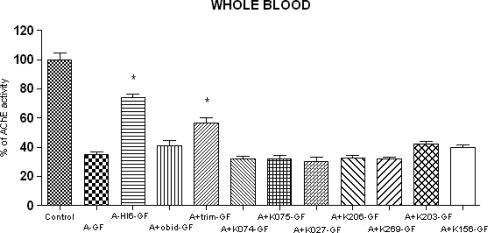
Changes of AChE activities in whole blood after cyclosarin (GF) intoxication and administration of AChE reactivators. The absolute value of control, where only atropine (A) was applied (100%) was 13.15 ± 0.881 μcat/ml. Values of results were expressed as mean ± SD, n = 6. AChE was inhibited on 35% of activity of control. The reactivation cyclosarin-inhibited AChE were found after administration of HI-6 (40%), obidoxime (6%), trimedoxime (22%), K203 (7%) and K156 (5%). The administration of other newly synthesized oximes was not effective. : K074 (−3%), K075 (−3%), K027 (−4%), K206 (−2%), K269 (−3%). The reactivation potency was compared to intoxicated group, treated only by atropine (A-GF). The significant changes in AChE activity were recorded between intoxicated group (A-GF) and groups treated by HI-6 and trimedoxime (p<0.001).

The baseline AChE activity in blood was 13.15 ± 0.881 μcat/mL. Cyclosarin intoxication produced strong depression of AChE activity in blood. The activity decreased approximately to the one third of original activity (33%). Among the currently available oximes, HI-6 (40%) was found to be the best reactivator of the cyclosarin-inhibited AChE. If other commercial oximes are considered, the better one seems to be trimedoxime (22%) followed obidoxime (6%). From the group of the newly synthesized oximes, the best result was found after administration of K203 (7%). All other oximes, except oxime K156 (5%) were ineffective. Results are summarized in Figure 3.

**Figure 4.**
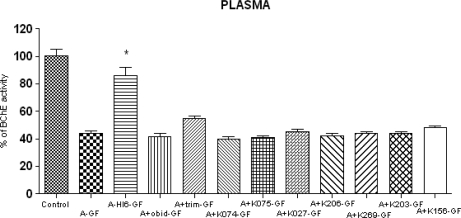
Changes of BChE activities in plasma after cyclosarin (GF) intoxication and administration of AChE reactivators. The absolute value of control, where only atropine (A) was applied (100%) was 1.253 ± 0.252 μcat/ml. Values of results were expressed as mean ± SD, n = 6. AChE was inhibited on 44% of activity of control. The reactivation cyclosarin-inhibited AChE were found after administration of HI-6 (42%), trimedoxime (11%), K027 (1%) and K156 (4%). The administration of other oximes was not effective: obidoxime (−1%), K074 (−4%), K075 (−3%), K206 (−2%), K269 (0%) and K203 (0%). The reactivation potency was compared to intoxicated group, treated only by atropine (A-GF). The significant change in AChE activity was recorded between intoxicated group (A-GF) and group treated by HI-6 (p<0.001).

The baseline BChE activity in plasma was 1.253 ± 0.252 μcat/mL. The BChE activity was also strongly decreased after cyclosarin intoxication. The residual activity of cyclosarin-inhibited BChE was 44 % in respect to original activity. From the currently available oximes, the oxime HI-6 (42%) achieved the best results again. Trimedoxime (11%) was also partially effective. All newly synthesized oximes were unable to reactivate cyclosarin-inhibited BChE and were as ineffective as the current commercially used obidoxime. Summarized results are shown in Figure 4.

**Figure 5.**
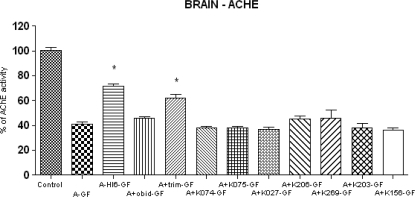
Changes of AChE activities in brain after cyclosarin (GF) intoxication and administration of AChE reactivators. The absolute value of control where only atropine (A) was applied (100%) was 95.20 ± 4.357 μcat/ml. Values of results were expressed as mean ± SD, n = 6. AChE was inhibited on 41% of activity of control. The reactivation cyclosarin-inhibited AChE were found after administration of HI-6 (31%), obidoxime (5%), trimedoxime (21%), K206 (4%) and K269 (5%). The administration of other newly synthesized oximes was not effective: K074 (−2%), K075 (−2%), K027 (−3%), K203 (−2%), K156 (−4%). The reactivation potency was compared to intoxicated group, treated only by atropine (A-GF). The significant changes in AChE activity were recorded between intoxicated group (A-GF) and groups treated by HI-6 and trimedoxime (p<0.001).

**Figure 6.**
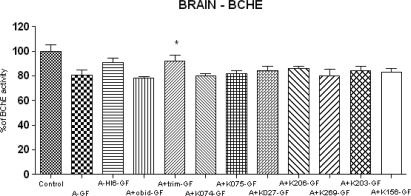
Changes of BChE activities in brain after cyclosarin (GF) intoxication and administration of AChE reactivators. The absolute value of control, where only atropine (A) was applied (100%) was 5.308 ± 0.474 μcat/ml. Values of results were expressed as mean ± SD, n = 6. AChE was inhibited on 81% of activity of control. The reactivation cyclosarin-inhibited AChE were found after administration of HI-6 (10%), trimedoxime (11%), K075 (1%), K027 (3%) and K206 (4%), K203 (3%) and K156 (2%). The administration of other oximes was not effective: obidoxime (−3%), K074 (−1%) and K269 (−1%). The reactivation potency was compared to intoxicated group, treated only by atropine (A-GF). The significant change in AChE activity was recorded between intoxicated group (A-GF) and group treated by trimedoxime (p<0.001).

The baseline AChE activity in brain was 95.20 ± 4.357 and BChE activity was 5.308 ± 0.474 μcat/ml. Strong inhibition of acetylcholinesterase (41%) was recorded in central nervous system (CNS). The results of all oximes are summarized in Figure 5 (AChE) and in Figure 6 (BChE). The inhibition of BChE (81%) was not so strong with respect to peripheral compartment (plasma). Only the oxime HI-6 (AChE 31%, BChE 10%) and trimedoxime (AChE 21%, BChE 11%) were able to partially protect cholinesterase in brain tissues. HI-6 seems to be the best reactivator again, because it was able to increase AChE activity for more than 30% in CNS. From the newly synthesized oximes only K206 and K269 were comparable with obidoxime (5%) reactivation potency.

Generally, the efficacy of ChE reactivators depends on their reactivity and affinity towards organophosphate-inhibited enzyme. Their reactivity is derived from the nucleophilic activity of oxime anion that is bound on pyridinium ring [15]. They differ from each other by the position of the oxime group on the pyridinium ring and linker between pyridinium rings. The affinity of oximes for intact enzyme is determined by various physicochemical factors such as electrostatic attraction and repulsion, hydrophobic interactions and by the shape and size of the whole molecule as well as the functional groups [21].

Bisquaternary reactivators have higher affinity towards both intact and inhibited ChE and higher potency to reactivate nerve agent-inhibited ChE compared to monoquaternary ones [15]. All used reactivators are bisquaternary oximes and the two quaternary nitrogens in their structures increases affinity of the oxime reactivators to the inhibited cholinesterases. Quaternary nitrogen binds reactivator, as same as acetylcholine does, to the anionic site of the enzyme [7].

The number and position of the oxime group(s) on the pyridinium ring is other factor influencing the reactivation efficacy. One oxime group in structure of AChE reactivator is necessary for the sufficient reactivation process [16]. More important than the number is position of the oxime group on the rings. From the *in vitro* tests the reactivators with the oxime group in the position 2 are the best reactivators of cyclosarin-inhibited AChE [7] and best reactivation potency of tabun-inhibited AChE was observed for oximes with the oxime group in that position [17,18]. Reactivators used in this comparative study, except HI-6, had oxime group in position 4. The results this *in vivo* study were compared with previously obtained *in vitro* results. None from the newly synthesized reactivators has better reactivation potency than the currently used HI-6 with its oxime group in position 2 on the pyridinium ring.

The linking chain between two pyridinium rings is another important factor influencing oximes’ potency. Although this part of oxime reactivator molecule does not play any role in the dephosphorylation process, it is a major factor influencing reactivation rates [19,20]. There is also only one difference between the obidoxime and trimedoxime connection chains (linkers). Obidoxime has oxygen in the linking chain. Obidoxime reactivation potency in both the peripheral and also the central compartment was worse than the reactivation efficacy of trimedoxime. Similar molecular structures are the newly synthesized oximes K027 (one oxime group is replaced by an amidic group) and K156 (there is only one oxime group in the molecule), but none of both oximes were able to reactivate cyclosarin-inhibited cholinesterases. It is clear, that presence of one oxime group is necessary for reactivation process. The presence of second one does not increase reactivation potency in the case of intoxication caused by tabun, but in cyclosarin-inhibited cholinesterases it can play positive role.

The other change is a double bond in chain (oximes K206, K269, K203 and K075). Double bonds make the linker more rigid and change the reactivator’s conformation [21]. This change in conformation can influence interaction between oxime and internal structure of enzyme. These oximes were ineffective in both compartments. The oxygen in the linker also can change oxime conformation. If we compare reactivation efficacy of obidoxime and trimedoxime, is clear that oxygen in the linker demonstrably decrease the reactivation potency [22].

The inhibitions of ChE in CNS after administration of cyclosarin were strong. The depression of AChE was stronger than inhibition of BChE. The reactivations of both ChE using newly synthesized oximes were not statistically significant. Only HI-6 and trimedoxime were able to partially protect the ChE in brain.

## 3. Experimental Section

### 3.1. Chemicals

The nerve agent cyclosarin (GF agent; *O*-cyclohexyl-*N,N*-methyl phosphonofluoridate) of 97% purity was obtained from Military Technical Institute of Protection (Brno, Czech Republic) and stored in glass ampoules (0.3 mL). Its solution for experiments was prepared immediately before use due to the known spontaneous hydrolysis during long term storage. All other chemicals were obtained from Sigma-Aldrich (Prague, Czech Republic). Oximes were synthesized at the Department of Toxicology, Faculty of Military Health Sciences (Hradec Kralove, Czech Republic). The purity of prepared oximes was approximately 96–99% [9–14].

### 3.2. Animals

Male Wistar rats, weighing from 180 to 200 g, were purchased from Anlab s.r.o. (Prague, Czech Republic). The animals were maintained in an air-conditioned room (the temperature were 22 ± 2 °C, the humidity was 50 ± 10%, with light from 7 a.m. to 7 p.m.), and were allowed free access to standard chow type SP 1, achieved from Velas s.r.o. (Prague, Czech Republic) and tap water. Housing of animals was realized in the Central Vivarium of Faculty of Military Health Sciences, Hradec Kralove. The experiment was performed under permission and supervision of the Ethic Committee of the Faculty of Military Health Sciences, Hradec Kralove.

### 3.3. Dosing and Sample Collection

Although the main routes of administration of organophosphorus nerve agents are percutaneous or inhalation, we used intramuscular administration (i.m.) in our experiments, because there is better comparability with already available data [23]. A single dose of 1 × LD_50_ (120 μg/kg) of cyclosarin was injected i.m.. Before each experiment, actual cyclosarin toxicity determinations were done to prove that the dose to be administered really corresponds to the 1 × LD_50_ dose. Cyclosarin-induced toxicity was evaluated by the assessment of LD_50_ values and their confidence limits that were calculated by probit analysis by deaths occurring within 24 hours after administration of the nerve agent at five different doses, with six rats per dose [24,25].

Oximes in therapeutic dosages (5 % LD_50_) (Table 1) in combination with dose of atropine (21 mg/kg) were administered i.m. 5 min before intoxication. First control group was treated only by dose of atropine and after 5 min was injected saline solution i.m. instead of nerve agent. The second control group of animals was also treated with a dose of atropine and 5 min after cyclosarin was administered.

Rats were anesthetized with CO_2_ and killed by decapitation 30 min after the nerve agent intoxication. The time interval, 30 min, of testing the reactivation potency of oximes was chosen based on our long-standing experiences. After decapitation, the trunk blood was collected in heparinized tubes and one part of this blood separated into plasma and erythrocytes by centrifugation (3000 × g for 15 min, 15°C) with Universal 320R (Hettich, Germany). In the whole blood, the ratio between AChE and BChE is approximately 95:5. The brains were removed from the skulls and stored at −80°C until the assay [26].

### 3.4. Biochemical Examinations

The whole blood was measured the day when the samples were collected. The blood samples were hemolyzed by using 0.02M Tris buffer, pH 7.6 (ratio: 1:20) for 5 min. Plasma samples were stored as other tissues until the assay (−80°C). After thawing, brains were homogenized (weight of tissue: volume - 1: 10; 0.02M Tris buffer, pH 7.6). Each sample was mixed with Ultra–Turrax homogenizer (Janke & Kunkel, Germany) for 20 seconds. Homogenates were used for enzymatic analysis. The activities of AChE and BChE were assessed by standard spectrofotometric Ellman’s method with acetylthiocholine or butyrylthiocholine iodides as substrates and 5,5‘-dithiobis(2-nitrobenzoic) acid as a chromogen [27] modified in wavelength 436 nm (because of influence caused by hemoglobin). The spectrophotometer Helios Alpha (Electron Corporation, Great Britain) was used for determination of absorbancy. The results were calculated as μcat/ml of homogenate, hemolysate or plasma.

### 3.5. Statistical Evaluation

The percent of reactivation R was taken as an outputting parameter. The value of the R was calculated according following equation:

(1) $$R=\frac{\Delta A_{r}-\Delta Ai}{\Delta A_{0}-\Delta Ai}\times 100(\%)$$

The symbol ΔA_0_ means absorbance provided by mixture with intact AChE (in this mixture was no inhibitor as well as no reactivator), ΔA_i_ is absorbance of mixture with inhibited AChE (no reactivator) and ΔA_r_ indicates AChE activity influenced by inhibitor and also reactivator [28]. The number of animals per group was six. Enzyme activities in tissue homogenates were expressed as the mean ± standard deviation (n=6). The ANOVA test was used for determination of statistical difference (Graph Pad Prism 4.0) (p<0.001).

## 4. Conclusions

In conclusion, the newly synthesized oximes were not able to surpass the efficacy of a currently used drug – HI-6. Only trimedoxime in both compartments was partially effective. Some newly synthesized oximes (K203; K156) were as effective as obidoxime in blood, which is not sufficient for treatment of cyclosarin-poisoning. No reactivation effect in central compartment was found after administration of all newly synthesized oximes. The partially protection of brain ChE was found only after administration of HI-6 and trimedoxime.

## Figures and Tables

**Table 1.** Oximes in therapeutic dosages corresponded to 5 % of LD_50_, these doses were administer to the animals.

**OXIME REACTIVATOR**	**DOSE CORRESPONING TO 5 % OF LD_50_**

**HI-6**	39.0 mg/kg
**Obidoxime**	10.5 mg/kg
**Trimedoxime**	7.5 mg/kg
**K206**	19.3 mg/kg
**K269**	5.7 mg/kg
**K203**	16.3 mg/kg
**K074**	23.0 mg/kg
**K075**	22.9 mg/kg
**K027**	22.3 mg/kg
**K156**	6.4 mg/kg
